# Development of One Pot Strategy for Hyper Production and In Vivo Evaluation of Lovastatin

**DOI:** 10.3390/molecules25194380

**Published:** 2020-09-24

**Authors:** Muhammad Azeem, Muhammad Arshad, Saqib Mahmood, Shazia Abrar, Ameer Fawad Zahoor, Sadia Javed, Bisma Tariq, Khizar Hayyat

**Affiliations:** 1 Department of Biochemistry, Government College University, Faisalabad 38000, Pakistan; azeem.fgs@gmail.com (M.A.); bismatariq@yahoo.com (B.T.); khizarhayyat61@gmail.com (K.H.); 2 Department of Basic Sciences, University of Veterinary & Animal Sciences, Jhang Campus, Jhang 35200, Pakistan; muhammad.arshad@uvas.edu.pk; 3 Department of Botany, Government College University, Faisalabad 38000, Pakistan; drsaqibj@gmail.com; 4 Department of Applied Chemistry, Government College University, Faisalabad 38000, Pakistan; shaziaabrar@gcuf.edu.pk; 5 Department of Chemistry, Government College University, Faisalabad 38000, Pakistan; fawad.zahoor@gmail.com

**Keywords:** lovastatin, pretreatment, mutant, *Aspergillus terreus*, rats, fermentation, hyper-cholesterolemia

## Abstract

The aim of this project was to improve the *Aspergillus terreus* strain and pretreatment of sugarcane bagasse as carrier substrate for bulk production of lovastatin, a cholesterol-lowering drug, in solid state fermentation. Sugarcane bagasse was treated with alkali (1–3% NaOH) for the conversion of complex polysaccharides into simple sugars for better utilization of carrier substrate by microorganism for maximum lovastatin production. Ethidium bromide (time of exposure 30–180 min) was used to induce mutation in *Aspergillus terreus* and the best mutant was selected on the basis of inhibition zone appeared on petri plates. Fermented lovastatin was quantified by high-performance liquid chromatography. The fermented lovastatin, produced by parent and mutant *Aspergillus terreus* strain, was checked on body weight, blood glucose and serum cholesterol, ALT, AST, HDL-C, LDL-C, TG and TC levels of rats for their cholesterol lowering capacity. Our results indicate that selected strain along with 2% NaOH treated sugar cane bagasse was best suitable for bulk production of lovastatin by fermentation and fermented lovastatin effectively lower the cholesterol level of rats.

## 1. Introduction

**Figure 1:**
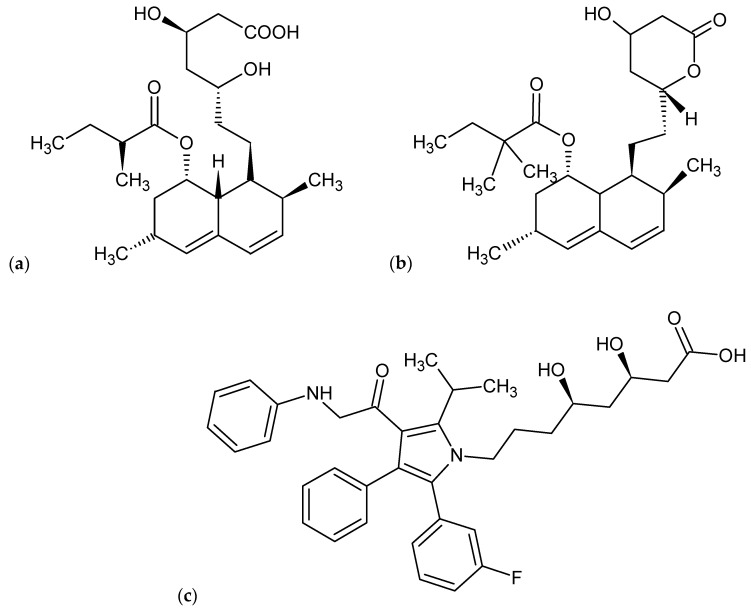
Structures of statins: (**a**) structure of lovastatin; (**b**) simvastatin; (**c**) atorvastatin.

Lovastatin belongs to an important class of statins and is frequently used as a cholesterol- lowering drug. Fungally-derived lovastatin is generally considered as a “safe drug”. *Aspergillus terreus* is the most reported microorganism for the production of lovastatin [1,2]. As compared with synthetic statins lovastatin (monacolin K) is more effective for hypercholesterolemic conditions as it has lipid lowering capacity in the serum [3]. Lovastatin is structurally similar to hydroxylmethyl glutarate (HMG), a part of HMG-CoA just like mevastatin, which is used as a substrate in cholesterol biosynthesis through the mevalonic acid pathway [4]. Lovastatin has 20,000-fold more binding affinity than HMG-CoA [2]. Lovastatin is usually activated by in vivo hydrolysis of its lactone ring. This is the main biologically effective form of lovastatin and it also has antifungal property. Simvastatin can be synthesized by acetylation of fermented lovastatin while atorvastatin is totally a synthetic hydroxymethylglutaryl coenzyme A (HMG-CoA) reductase inhibitor (Figure 1a–c).

**Figure 2:**
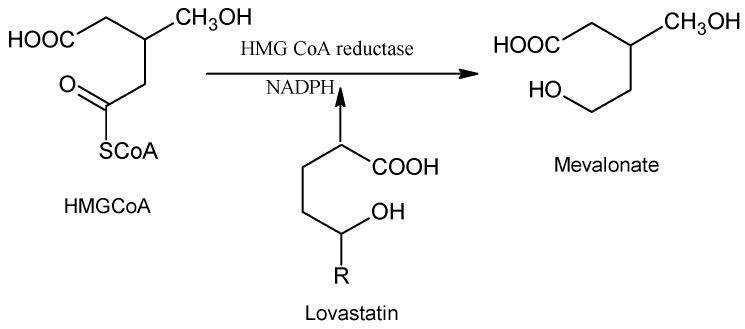
Mechanism of action of lovastatin.

The lactone ring after hydrolysis has structural similarity to the tetrahedral intermediate synthesized by reductase to bind the drug with HMG-CoA reductase [4]. The double ring of lovastatin attaches to coenzyme A in the active site. This process inhibits mevalonate synthesis which leads to inhibition of cholesterol production in the body (Figure 2). This causes a reduction of LDL cholesterol in serum and triglyceride, with elevation in HDL cholesterol [5]. Lovastatin has also been considered a promising compound in the treatment of the pathophysiology of fragile X syndrome [6]. Hyper production of various useful products by microbial culture is the main objective nowadays. Certain processes for instance, mutagenesis, selection and genetic engineering are being used for the hyper production of certain useful products by improving microbial strains [7]. A mutagenic treatment is also a useful method for high productivity of important industrial products [8].

Pretreatment of lignocellulosic biomass is a very effective way of degrading biomass into simpler molecules before enzymatic hydrolysis. There are few effective pretreatments that significantly enable cellulosic fraction of lignocellulosic biomass cell wall for enzymatic hydrolysis of cellulosic degradation into simpler monomers molecules [7,8]. Acid-base pretreatment is the most efficient method for degradation and removal of lignin and hemicellulose from sugarcane bagasse and gives high amounts of fermentable sugar on enzymatic hydrolysis [9,10,11]. Therefore, the present study was planned with the main objective to develop a hyper producing mutant of *Aspergillus terreus* and hydrolysis of complex polysaccharide of sugarcane bagasse by alkali treatment for bulk production of and comparison of both strains of *Aspergillus terreus*; parent and chemically improved along with synthetic atorvastatin to estimate their hypocholesterolemic efficiency in rats.

## 2. Results and Discussion

In the present study the compositional analysis of sugarcane bagasse revealed that sugarcane bagasse consists of 42% cellulose, 21.25% hemicelluloses, 22.76% lignin, 2.97% total sugars, 7.19% reducing sugars, 0.86% ash and 7.3% moisture content. However, Javed et al., [12] found 40% cellulose, 21% hemicelluloses, 23% lignin and 0.9% ash contents in sugarcane bagasse. Moreover, Rocha et al. [13] reported 21.1% lignin, 27% hemicellulose, 45.5% cellulose and 2.2% ash in sugarcane bagasse while Rabelo et al. [14] revealed 25% lignin, 23.2% hemicellulose, 38.4% cellulose, and 1.5% ash in sugarcane bagasse. To facilitate the rapid and efficient hydrolysis and conversion of carbohydrates to fermentable sugars it is necessary to hydrolyze the biomass into macroscopic and microscopic size and its submicroscopic structural and chemical composition [15]. Cell wall is made up of complex polysaccharides including lignin, cellulose and hemicellulose that limit the availability of substrate due to shielding effect of these polysaccharides. Chemical and enzyme mediated pretreatments disrupt the sugarcane bagasse cell wall by degradation of the lignin, hemicellulose and cellulose. NaOH breaks the α-aryl ester bonds of polyphenolic monomers and weakens the H-bonds that promote the swelling of cellulose [16].

**Figure 3:**
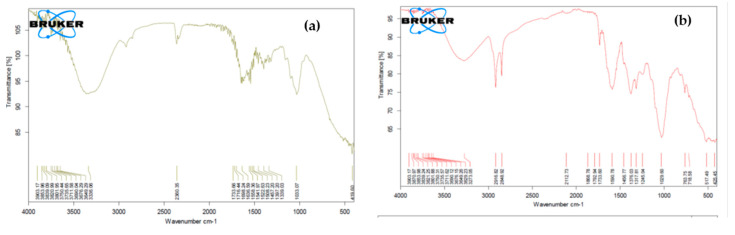

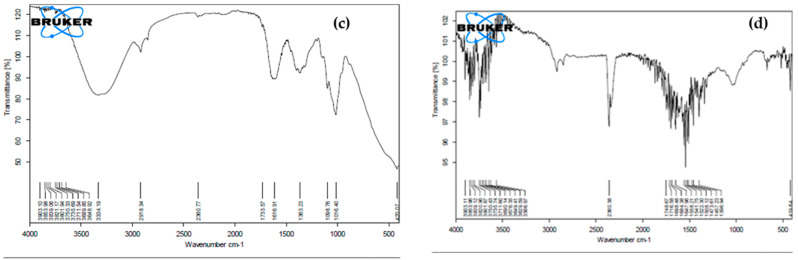
Fourier Transform Infrared Spectroscopy (FTIR) for bagasse (**a**) native (**b**) treated with 1% NaOH (**c**) 2% NaOH (**d**) 3% NaOH.

A comparison of the Fourier transform infrared (FTIR) spectra of native and NaOH treated sugarcane bagasse is shown in Figure 3a–d. Figure 3a indicates the region around 1.247 cm^−1^ was characteristic of hemicellulose and lignin due to the stretching of C-O bond [17]. A band around 1.457 cm^−1^ has shown a deformation of lignin CH_2_ and CH_3_, and band around 1.508 cm^−1^ represents the C=C stretching of the aromatic ring in lignin [18,19]. The band around 1.636 cm^−1^ exhibits the stretching of the C=O and C=C lignin aromatic rings. A band around 1.733 cm^−1^ is characteristic of C=O stretching of unconjugated hemicellulose. The region between 3.801 and 3.676 cm^−1^ indicates the crystalline structure of cellulose. This region constitutes the sum of the vibration of valence bands of the H-bond of the OH group and the bands of intramolecular and intermolecular H-bonds [20]. Figure 3a,b,d show the FTIR spectrum of 1%, 2% and 3% alkali-treated sugarcane bagasse, respectively. The region between 1.300 and 1.700 cm^−1^ clearly shows peaks after alkali hydrolysis indicating the removal of hemicellulose. In the region of 1.245 cm^−1^, the removal of hemicellulose is also evident. 

Alkaline hydrolysis promotes removal of lignin moieties. The lignin macromolecule regions around 1.868 cm^−1^, 1.733 cm^−1^, and 1.580 cm^−1^ and cellulose-containing regions around 2.918 cm^−1^ and 2.850 cm^−1^ have shown noticeable changes after alkaline hydrolysis. It is evident from these changes that a delignification process has occurred in the sugarcane bagasse.

**Figure 4:**
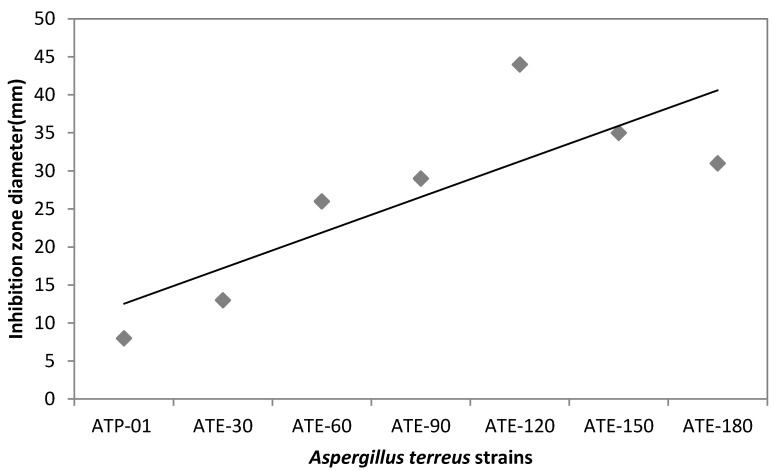
Inhibition zone diameter on plate vs. *Aspergillus terreus* parent and mutant strains. ATP-01: *Aspergillus terreus* parent strain; ATE: *Aspergillus terreus* treated with ethidium bromide with various time of exposures (30–180 min).

From ethidium bromide (EB) treated plates, 10 hyperproducing mutants were selected on the basis of their efficiency on plates. A total of 10 positive mutants were picked predominantly on the basis of their bigger zone of inhibition on *Candida albicans* grown on agar plates (Figure 4). The mutant ATE-120 obtained at 120 min dose of EB manifested 44 mm clearance zone (Figure 4). 

These mutants were also evaluated through still culture flask experiments. The mutants of *Aspergillus terreus* along with parent strain were used for production, using sugarcane bagasse in solid state fermentation (SSF). Among these, the maximum production (91 ± 1.77 mg/L) with dry cell mass (4.49 ± 0.81 g/L) was noticed for the mutant ATE-120 using 2% NaOH treated sugarcane bagasse (Table 1). The results indicated that ATE-120 was the most hyper-active mutant, producing more lovastatin over the parent strain (23 ± 1.54 mg/L) in similar culture conditions. Ferron et al. [21] reported a positive correlation between the lovastatin titer obtained in liquid cultures of the isolate and the measured diameter of inhibition zone on agar which is in accordance with our work. Samiee et al. [22] also noticed that *Aspergillus terreus* is a best lovastatin (55 mg/L) producing strain out of 110 fungal strains. Mangunwardoyo et al. [23] detected the highest lovastatin production (85.88 mg/L) by *Aspergillus flavus* UICC 360. Moreover, Lakshman and Radha [2] find out a maximum lovastatin concentration (113 μg/mL) by *P. ostreatus*.

Besides, the blood glucose levels of high cholesterol dietfor fermented parent derived lovastatin (PL), mutant derived lovastatin (ML), and atorvastatin groups were crucially higher than in control group during the whole experimental period after administration of high cholesterol diet in rats (Table 2). Concentrations of serum cholesterol in rats fed on high cholesterol diet rats were slightly higher than those in normal control rats. It was also observed that cholesterol level was notably lower in rats treated with parent, mutant derived lovastatin and atorvastatin than in high cholesterol diet (HCD) group at 20 and 60 days. Changes in body and kidney weights in the five groups of rats are shown in Table 1. Moreover, the reactive oxygen species (ROS), developed in response to increase in blood glucose and lipid levels, are the main causative agents for liver damage [24].

The findings of the present study also demonstrated that the serum aspartate aminotransferase (AST) and alanine aminotransferase (ALT) were remarkably increased in high cholesterol diet (HCD) than normal and fermented lovastatin (PL & ML) diet. Serum total cholesterol (TC), triglycerides (TG) and low density lipoproteins (LDL) levels were also notably increased after 20, 40 and 60 days. Thus, HDL-cholesterol level reduced in high cholesterol diet (HCD) group as compared to normal rats. These findings are in close agreement with the Stepherd [25] work who found the inhibition of HMG-CoA reductase by a class of drug statins in rat liver. The reason behind increase in LDL-cholesterol concentration might be the reduction in receptors or reduced low density lipoproteins (LDL) binding to its receptor [26]. This change in receptor is responsible for the elevation of blood cholesterol level, in response to high cholesterol diet (HCD). In the present study, fermented lovastatin (PL and ML) and synthetic atorvastatin reduced the total cholesterol (TC), triglycerides (TG) and low density lipoproteins (LDL) levels and increased the high dentistry lipoproteins (HDL) level (Table 3). Low level of high density lipoproteins (HDL) may develop atherosclerosis because high density lipoproteins (HDL) absorb cholesterol and transport it to the liver for metabolism. In hypocholesterolemic rats, decreased HDL level may be due to reduced activity of lipoprotein lipase (LPL). LPL is a water-soluble enzyme that catalyzes the hydrolysis of triglycerides (TGs) into lipoproteins. A decrease in lipoprotein lipase activity is associated with an increase and decrease in high density lipoprotein (HDL) and plasma triglycerides (TGs) respectively.

## 3. Material and Methods

### 3.1. Pretreatment of Substrate

Sugarcane bagasse was obtained from Shakarganj Sugars Mills Jhang (Jhang, Pakistan. The sugarcane bagasse was washed thoroughly with cold water to remove dust and was ground to 40 mm. Thirty mL samples of NaOH with different concentrations (1–3%) were made and 2 g of powdered bagasse was added to each concentration. These samples were kept at 80 °C and neutral pH in microwave for 10 min then filtered and rinsed with tap water. The pretreated samples were dried at 65 °C [9]. The native and treated sugarcane bagasse samples were analyzed using Fourier Transform Infrared Spectroscopy (FTIR) by the method of Chandel et al., [11]. The spectra were collected in the range 4000 to 500 cm^−1^ with a resolution of 4 cm^−1^ per minute and room temperature detector (Tensor II FTIR, Bruker, Bremen, Germany).

### 3.2. Mutagenic Treatments

Ethidium bromide was used to induce mutation in the *Aspergillus terreus* strain. A stock solution of 0.5 mg/mL ethidium bromide (*w*/*v*) was prepared. The stock solution of EB (Sigma-Aldrich, Gillingham, UK) was prepared. It was mixed with Vogel medium (9 mL), which contained 1 × 107 spores mL^−1^ of *Aspergillus terreus* and kept it in a water bath (Eyela, Tokyo, Japan) at 37 °C. One milliliter sample was withdrawn after a specific time interval, and cell pellet was washed thrice with normal saline solution. The sample was centrifuged at 12,000 rpm for 1 min using centrifuge machine (Mikro 20 Hettich, Tuttlingen, Germany), to separate out the mutagen from sample [9].

### 3.3. Selection of Best Mutant

*Candida albicans* was used for the selection of best mutant. It was grown on PDA dishes at 28 °C for 12 h. 50 µL of fermented lovastatin extract were added onto a 6 mm diameter paper disk. These paper disks were placed on the surface of a 90 mm diameter *C. albicans* plate. The maximum distance of 15mm was kept between lovastatin impregnated disks on a plate. The plates were incubated for 6 h and zones of inhibition were recorded. A large diameter of the inhibition zone implied a high titer of lovastatin [21].

### 3.4. Lovastatin Production

Lovastatin was produced in solid state fermentation (SSF) by the method as described by Javed et al. [9]. Experiments were performed using 500 mL Erlenmeyer flasks in a temperature-controlled incubator (SLI-600ND, Eyela). Two g of sugar cane bagasse as a carrier substrate was taken in triplicate 500 mL Erlenmeyer flasks moistened with 7 mL Vogel medium. pH was adjusted to 6 using M NaOH/M HCl solutions and SSF media flasks were sterilized (121 °C) in autoclave for 15 min. After cooling to room temperature, the triplicate flasks were inoculated with 5 mL of homogenous spore suspension of *Aspergillus terreus* and the flasks were subjected to SSF under still culture conditions for 72 h.

### 3.5. Extraction of Lovastatin

10% 1N HCl was added to the fermentation broth after 3 days of fermentation. The acidified broth was extracted with equal volume of ethyl acetate at 70 °C temperature and 180 rpm for 2 h. The Whatman filter paper No. 40 was used for the filtration of broth. The filtrate was centrifuged at 3000 rpm for ten mins and the organic phase was collected. 10 mL of 1% trifluoroacetic acid was mixed in the one ml of organic phase of filtrate for lactonization. The extract was heated at 80 °C to evaporate the moisture, diluted with one mL acetonitrile and filtered for high-HPLC analysis.

### 3.6. Analysis of Lovastatin

The identification and quantification of lovastatin was carried out by HPLC method [27]. The samples were prepared by 10 fold diluting the filtered broth with acetonitrile-water (1:1 by volume) and analyzed using an HPLC (Hitachi, Ibaraki, Japan) equipped with a UV detector (Hitachi L-2400) at 238 nm and a Hitachi L-2130 (C-18) column. The solvent was prepared by mixing acetonitrile and 0.1% phosphoric acid (60:40 by volume). A 20 μL sample was injected with a flow rate of 1.5 mL min^−1^.

### 3.7. In Vivo Application of Lovastatin

Thirty male albino rats were selected for the induction of lovastatin. These male rats of 200–230 g of weight were obtained from National Institute of Health (Islamabad, Pakistan). These albino rats were group into 5 groups each containing 6 rats and caged into separate cages under suitable temperature of 25 ± 5 °C. Proper ventilation system is maintained and standard feed and tap water is provided according to their weight. According to the method explained by Kamal and Thanaa [26] with high cholesterol diet was prepared. 20 mg/day lovastatin dose was administered to rats, fed on high cholesterol diet. Five groups were made according to their diet:

Group 1 contains normal rats on normal diet
Group 2 contains rats with high cholesterol diet (HCD)
Group 3 HCD+ fermented lovastatin derived from parent *Aspergillus terreus* strain (PL)
Group 4 HCD+ fermented lovastatin derived from mutant *Aspergillus terreus* strain (ML)
Group 5 contains rats which are on synthetic statin

The efficacy of fermented lovastatin on serum of rats AST, HDL-C, ALT, LDL-C, TC and TG level of rats was determined by the method of Kamal and Thanaa, [26] and Javed et al. [9]. Concentration of serum cholesterol was determined by a Cholesterol-E kit. Blood glucose level was determined by a standard kit method.

### 3.8. Statistical Analysis

Statistical significance of the differences between mean values was calculated by ANOVA under CRD and DMR test using Minitab 2000 version 13.2 statistical software (Minitab Inc., State College, PA, USA). A probability value of *p* ≤ 0.05 was considered to denote a statistically significant difference [28].

## 4. Conclusions

It can be inferred from the present study that strain improvement of *Aspergillus terreus* using ethidium bromide and pretreatment of sugarcane bagasse using NaOH for hydrolysis of complex polysaccharides for better utilization of substrate by fungal strain were effective strategies to hyper- produce lovastatin and successfully treat hypercholesterolemia. The present data would certainly help to ascertain the ability of fermented lovastatin as the potential drug for hypercholesterolemia to be used in the pharmaceutical industry.

## Figures and Tables

**Table 1:** Production of lovastatin by various strains growing over native and treated substrates.

Strains	Native		1% NaOH		2% NaOH		3% NaOH	
	Lovastatin (mg/L)	Biomass (g)	Lovastatin (mg/L)	Biomass (g)	Lovastatin (mg/L)	Biomass (g)	Lovastatin (mg/L)	Biomass (mg/L)
**Parent**	8.35 ± 0.56	4.16 ± 0.11	18.31 ± 0.96	3.28 ± 0.11	20.28 ± 0.69	3.81 ± 0.67	34.54 ± 0.61	4.22 ± 0.59
ATE-30	11.28 ± 0.56	4.28 ± 0.11	33.43 ± 1.17	3.28 ± 0.14	34.17 ± 0.33	3.27 ± 0.17	37.16 ± 1.11	4.33 ± 0.23
ATE-60	15.72 ± 5.15	3.66 ± 0.21	21.33 ± 1.01	4.21 ± 0.33	46.33 ± 0.56	4.31 ± 0.41	39.11 ± 1.34	3.58 ± 0.27
ATE-90	11.67 ± 1.01	4.67 ± 0.33	48.51 ± 1.22	4.61 ± 0.51	51.37 ± 0.81	4.56 ± 0.61	45.65 ± 1.30	4.13 ± 0.32
ATE-120	16.32 ± 0.97	3.84 ± 0.55	52.14 ± 1.31	4.88 ± 0.41	91 ± 1.77	4.49 ± 0.27	51.12. ± 1.61	4.66 ± 0.31
ATE-150	12.31 ± 1.21	3.54 ± 0.43	44.45 ± 1.12	3.91 ± 0.31	74.76 ± 1.22	3.64 ± 0.34	53.31 ± 1.51	4.31 ± 0.25
ATE-180	13.32 ± 0.79	4.20 ± 0.34	39.18 ± 0.97	3.87 ± 0.51	64.10 ± 1.22	4.12 ± 0.53	49.28 ± 1.32	4.56 ± 0.35

**Table 2:** Effect of fermented lovastatin on body weight, blood glucose and serum cholesterol levels in rats.

Groups	Days	Body Weight (g)	Blood Glucose Level (mg/dL)	Serum Cholesterol Level (mg/dL)
**Control**	20	230 ± 10	160 ± 12	63 ± 5
	40	265 ± 13	186 ± 11	61 ± 3
	60	281 ± 14	180 ± 15	58 ± 5
**HCD**	20	221 ± 11	470 ± 12	75 ± 4
	40	245 ± 15	510 ± 13	70 ± 7
	60	274 ± 18	540 ± 11	63 ± 6
**HCD-PL**	20	215 ± 15	430 ± 10	65 ± 10
	40	232 ± 10	390 ± 9	61 ± 3
	60	250 ± 13	425 ± 14	68 ± 4
**HCD-ML**	20	213 ± 9	410 ± 15	55 ± 5
	40	235 ± 13	360 ± 13	62 ± 3
	60	250 ± 16	350 ± 11	58 ± 5
**HCD-SA**	20	220 ± 17	445 ± 14	60 ± 6
	40	231 ± 12	418 ± 12	65 ± 7
	60	234 ± 14	390 ± 10	67 ± 4

Values (Mean ± SD) are average of six samples, Analyzed individually in triplicate (n = 1 × 3 × 3) *p* ≤ 0.05. HCD: High Cholesterol diet. HCD-PL: High cholesterol diet and parent strain derived lovastatin. HCD-ML: High Cholesterol diet and mutant strain derived lovastatin. HCD-SS: High cholesterol diet and synthetic atorvastatin.

**Table 3:** Effect of fermented lovastatin on lipid profile in rats.

Groups	Days	AST (mmol/L)	ALT (mmol/L)	HDL-C (mmol/L)	LDL-C (mmol/L)	TG (mmol/L)	TC (mmol/L)	%TC/HDL-C
**Control**	20	188 ± 8.44	62 ± 4.44	0.71 ± 0.03	0.50 ± 0.02	1.3 ± 0.01	1.82 ± 0.04	2.5
	40	182 ± 10.11	60 ± 3.11	0.78 ± 0.05	0.48 ± 0.03	1.24 ± 0.03	1.75 ± 0.03	2.2
	60	183 ± 7.90	61 ± 8.16	0.75 ± 0.09	0.50 ± 0.05	1.4 ± 0.04	1.8 ± 0.00	2.4
**HCD**	20	240 ± 11.21	58 ± 7.32	0.38 ± 0.04	7.45 ± 0.91	1.5 ± 0.02	7.3 ± 0.12	19.2
	40	236 ± 9.38	57 ± 5.87	0.36 ± 0.01	7.98 ± 0.24	1.57 ± 0.05	6.88 ± 0.08	19.1
	60	235 ± 8.47	59 ± 5.44	0.35 ± 0.03	8.84 ± 0.17	1.6 ± 0.06	6.2 ± 0.14	17.7
**HCD-PL**	20	209 ± 10.11	61 ± 6.10	0.50 ± 0.01	6.54 ± 0.65	1.75 ± 0.04	4.2 ± 0.11	8.4
	40	203 ± 6.38	62 ± 6.88	0.51 ± 0.05	5.63 ± 0.41	1.6 ± 0.05	4.8 ± 0.08	9.4
	60	204 ± 9.19	59 ± 6.54	0.53 ± 0.09	5.87 ± 0.33	1.7 ± 0.04	4.5 ± 0.09	8.5
**HCD-ML**	20	195 ± 11.32	64 ± 6.21	0.61 ± 0.07	4.32 ± 0.90	1.77 ± 0.08	3.45 ± 0.07	5.6
	40	190 ± 9.67	65 ± 8.44	0.62 ± 0.06	4.18 ± 0.46	1.8 ± 0.06	3.1 ± 0.01	5
	60	191 ± 12.21	64 ± 6.11	0.65 ± 0.05	3.87 ± 0.16	1.64 ± 0.01	3.87 ± 0.12	5.9
**HCD-SS**	20	208 ± 12.80	53 ± 5.19	0.50 ± 0.02	6.81 ± 0.80	0.9 ± 0.06	5.4 ± 0.90	10.8
	40	206 ± 9.73	55 ± 2.08	0.49 ± 0.07	6.13 ± 0.73	0.8 ± 0.07	5.2 ± 0.13	10.6
	60	207 ± 11.61	51 ± 4.17	0.47 ± 0.05	5.81 ± 0.65	1.1 ± 0.05	5 ± 0.15	10.6

Values (Mean ± SD) are average of six samples, Analyzed individually in triplicate (n = 1 × 3 × 3) *p* ≤ 0.05. HCD: High Cholesterol diet. HCD-PL: High cholesterol diet and parent strain derived lovastatin. HCD-ML: High Cholesterol diet and mutant strain derived lovastatin. HCD-SS: High cholesterol diet and synthetic atorvastatin. Aspartate aminotransferase (AST). Alanine aminotransferase (ALT). High density lipoproteins cholesterol (HDL-C). Low density lipoproteins cholesterol (LDL-C). Triglycerides (TC). Total cholesterol (TC).
